# Long-term effect of increasing water intake on repeated self-assessed health-related quality of life (HRQoL) in autosomal dominant polycystic kidney disease

**DOI:** 10.1093/ckj/sfae159

**Published:** 2024-06-07

**Authors:** Gopala Rangan, Margaret Allman-Farinelli, Neil Boudville, Mangalee Fernando, Imad Haloob, David C H Harris, Carmel M Hawley, Karthik Kumar, David W Johnson, Vincent W Lee, Jun Mai, Anna Rangan, Simon D Roger, Priyanka Sagar, Kamal Sud, Vicente Torres, Eswari Vilayur

**Affiliations:** Centre for Transplant and Renal Research, Westmead Institute for Medical Research, The University of Sydney, Sydney, Australia; Department of Renal Medicine, Westmead Hospital, Western Sydney Local Health District, Sydney, Australia; School of Nursing, The University of Sydney, Sydney, NSW, Australia; Sir Charles Gairdner Hospital, Perth, Australia; Medical School, University of Western Australia, Perth, Australia; Department of Renal Medicine, Prince of Wales Hospital, Sydney, NSW, Australia; Department of Renal Medicine, Bathurst Hospital, Bathurst, Australia; Centre for Transplant and Renal Research, Westmead Institute for Medical Research, The University of Sydney, Sydney, Australia; Department of Renal Medicine, Westmead Hospital, Western Sydney Local Health District, Sydney, Australia; Australasian Kidney Trials Network, University of Queensland at Princess Alexandra Hospital, Brisbane, Australia; Translational Research Institute, Brisbane, Australia; Department of Kidney and Transplant Services, Princess Alexandra Hospital, Brisbane, Australia; Gosford Nephrology, Gosford, Australia; Australasian Kidney Trials Network, University of Queensland at Princess Alexandra Hospital, Brisbane, Australia; Translational Research Institute, Brisbane, Australia; Department of Kidney and Transplant Services, Princess Alexandra Hospital, Brisbane, Australia; Centre for Transplant and Renal Research, Westmead Institute for Medical Research, The University of Sydney, Sydney, Australia; Department of Renal Medicine, Westmead Hospital, Western Sydney Local Health District, Sydney, Australia; Department of Renal Medicine, Liverpool Hospital, Southwestern Sydney Local Health District, Sydney, NSW, Australia; School of Nursing, The University of Sydney, Sydney, NSW, Australia; Renal Research, Gosford, Australia; Nepean Kidney Research Centre, Department of Renal Medicine, Nepean Hospital and Nepean Clinical School, The University of Sydney, Sydney, Australia; Nepean Kidney Research Centre, Department of Renal Medicine, Nepean Hospital and Nepean Clinical School, The University of Sydney, Sydney, Australia; Translational Polycystic Kidney Disease Center, Mayo Clinic, Rochester, USA; Department of Nephrology, John Hunter Hospital, Newcastle, Australia

**Keywords:** ADPKD, CKD, clinical trial, polycystic kidney disease, quality of life

## Abstract

**Background:**

The aim of this study was to determine the long-term effect of increasing water intake in patients with autosomal dominant polycystic kidney disease (ADPKD) on longitudinal changes in health-related quality of life (HRQoL) in the setting of a clinical trial.

**Methods:**

Self-completed HRQoL (using the KDQoL-SF, v.1.3 questionnaire) was assessed annually in participants of a 3-year randomized controlled clinical trial (*n* = 187), allocated (1:1) either to increase water intake to reduce urine osmolality to ≤270 mosmol/kg (implemented by dietetic coaching, self-monitoring tools, text messaging) or continue usual water intake.

**Results:**

Overall, 96% and 81.8% of participants (*n* = 187) completed the questionnaire at the baseline and final study visits, respectively. At baseline, the physical component summary score (PCS) and mental component summary score (MCS) were similar in the two groups (*P *> 0.05) and the five dimensions with the lowest scores in both groups were: energy and fatigue; general and overall health; sleep; emotional well-being; and pain. Within each group, there were no longitudinal changes over time. At the final visit, the PCS was higher in the increased water intake group (51.3 ± 7.6, mean ± standard deviation) compared to the usual water intake group 48.8 ± 9.3; *P *= 0.037) whereas the MCS was numerically similar. The improvement in the PCS was due to higher sub-scale values for physical functioning and pain (both *P *< 0.05). By multivariate analysis, only baseline PCS and height-corrected total kidney volume were associated with the final PCS (*P *< 0.05).

**Conclusion:**

HRQoL scores remained stable over a 3 year period, and were not adversely affected by the intervention to increase water intake. Future studies should evaluate the clinical significance of the higher PCS in the increased water intake group.

KEY LEARNING POINTS **What was known**:

- Increasing water intake to maintain hydration is widely suggested in clinical practice as an adjunct to slow the progression of autosomal dominant polycystic kidney but the long-term effects of health-related quality of life have not been evaluated in this population.

**This study adds**:

- The results showed that health-related quality of life remained stable over the 3 year period and was not detrimentally affected by increasing water intake group. Moreover, endpoints for physical functioning and pain improved with increased water intake.

**Potential impact**:

- This is the first long-term study to evaluate the role of increased water intake of health-related quality of life. This study suggests that advising patients to increase water intake does not have any detrimental effects on quality of life and may have positive benefits on pain and physical functioning, which should be investigated in future studies.

## INTRODUCTION

Autosomal dominant polycystic disease (ADPKD) is a chronic progressive cystic kidney disease that leads to kidney failure in ∼60% of affected individuals by the age of 60 years [1]. It is the most common genetic cause of kidney failure and is due to heterozygous germline mutations in *PKD1, PKD2* or rarely other genes [2]. It is noteworthy that even when kidney function is well preserved (estimated glomerular filtration rate >60 ml/min/1.73 m^2^), 88% of patients experience non-specific symptoms, such pain, fatigue, anxiety and malaise in addition to traditional disease manifestations such as hypertension [3, 4]. With age and disease progression, the impacts of kidney function decline and nephromegaly dominate the physical burden of disease together with a risk of worsening mental health and reduced health-related quality of life (HRQoL) [3].

Arginine vasopressin (AVP) is the most important postnatal growth factor for kidney cysts in ADPKD [5, 6]. Previous pivotal clinical trials have demonstrated that tolvaptan (a vasopressin receptor antagonist) slows kidney cyst growth and the decline in kidney function without affecting HRQoL in the short term [7–9]. It has been hypothesized that maintaining adequate hydration could also reduce kidney cyst growth by attenuating the release of AVP [10]. However, the PREVENT-ADPKD randomized controlled trial showed that increasing water intake to reduce 24 hour urine osmolality ≤270 mOsmol/kg (achieving a urine output of 3.0 vs. 2.3 l/day in the usual water intake group) for 3 years did not reduce the progression of kidney cyst growth or estimated glomerular filtration rate (eGFR) decline in ADPKD (stages 1–3 chronic kidney disease) [11, 12]. The intervention in the PREVENT-ADPKD trial was resource-intensive and required behavioural changes by participants to modify both daily fluid and dietary solute intake [11, 12]. In this regard, the proportion of patients in the intervention group stating that water intake could not be tolerated life-long, increased from 9% at 6 months to 21% at 3 years [11, 12]. The intervention in the PREVENT-ADPKD trial was implemented by regular coaching, SMS texting, and self-monitoring [11, 12]. Whereas closer contact with the intervention group could have had positive effects on well-being, it may have also led to internal discontent when participants did nor attain planned goals [13]. Chronic diseases and medical interventions may negatively influence physical, social and mental health of an individual and decrease quality of life [14, 15]. It is not clear whether the intervention to increase water intake could have reduced quality of life and overall motivation to continue therapeutic interventions [16].

The aim of the current study was to evaluate the effect of increasing water intake on quality of life in patients with ADPKD by comparing the intervention group with the usual water intake group in the PREVENT-ADPKD trial over the 3 years [11]. The specific aims were to first evaluate any longitudinal changes in HrQoL over time in the two groups, and second to assess inter-group differences at the baseline and the final study visits.

## MATERIALS AND METHODS

### Study population

The study design and primary results of the PREVENT-ADPKD trial have been described in previous publications [11, 12]. Briefly, a total of 187 patients with ADPKD, aged between 18 to 67 years with eGFR >30 ml/min/1.73 m^2^ were recruited from 13 Australian study centres, randomized to two groups, and asked to either maintain their usual fluid intake (Group A) or drink a prescribed increase in water intake (which was according to the free water clearance formula) (Group B) [11]. The primary endpoints of the trial were changes in disease-specific biomarkers [height-corrected total kidney volume (TKV)/Mayo subclass and eGFR] from baseline until 3 years [11].

### Procedure

All randomized participants were included in the analysis. HRQoL data were collected immediately following randomization (second study visit), annually and at the final study visit as pre-specified in the study protocol [11]. Participants were given a paper copy of the HRQoL instrument at the end of each study visit and told that the questionnaire was a validated tool to evaluate functional and psychological state. Participants self-completed the questionnaire but study staff were available during completion to provide any clarifications.

### Assessment of health-related quality of life

The Kidney Disease Quality of Life-Short-Form (KDQOL-SF) 1.3, developed by the Research and Development (RAND) Corporation, combines the generic Short-Form-36 (SF-36) and a kidney disease-specific instrument, and has been widely used in multiple studies of chronic kidney disease, including those with ADPKD [17–19]. The SF-36 has been used in national surveys of the general population and provides data on eight sub-scale dimensions (derived from 36 items) of health that include physical functioning (PF) (the extent to which a person is limited by their health in performing a range of physical functioning), role-physical (RP) (the effects of physical health on an individual's performance in their work or other daily activities), bodily pain (BP) (the severity of pain and the extent to which it might interfere with normal activities), general health (GH) (measure of self-assessed health status that includes indicators of current expectations and perception of health relative to that of others), vitality (VT) (defined as an individual's levels of energy and fatigue), social functioning (SF) (measures the impact of perceived health or emotional problems on the quality and quantity of social activities), role-emotional (RE) (measures the effects of emotional problems on an individual's work or other daily activities), and mental health (MH) (measures the amount of time an individual experienced nervousness, depression, anxiety or happiness). With the exception of two dimensions (PF and GH), the responses were related to the 4 weeks before completing the questionnaire. The SF-36 scores were aggregated into a physical component summary score (PCS) that reflected PF, RP, BP, and GH and a mental component summary score (MCS) that represented VT, SF, RE, and MH. The kidney-specific component included 11 sub-scales related to kidney disease-specific instrument (symptoms/problems, effects of kidney disease, burden of kidney disease, work status, cognitive function, quality of social interaction, sexual function, sleep, and social support). Since participants in the PREVENT-ADPKD trial had stages 1 to 3 chronic kidney disease, participants were asked to omit one question from the KDQOL-SF1.3 related to dialysis [11].

### Statistical analysis

Data from the KDQOL-SF1.3 were entered in a purpose-built excel template downloaded from the RAND corporation, and responses were weighted and transformed to scores ranging from 0 to 100, with higher scores indicating better self-assessed HRQoL. Data from the excel spreadsheet were then extracted and analysed using JMP Statistical software. The data had non-normal distribution using the Shapiro–Wilk test, and continuous variables for descriptive statistics were expressed as mean (standard deviation) and median (quartile 1 to quartile 3). The non-parametric Kruskal–Wallis one-way ANOVA and Wilcoxon ranked sums test were used to determine comparisons between groups. To evaluate the first aim of the study (longitudinal changes in HRQoL over time), data were analysed by ANOVA, and if statistical differences were identified, correction for multiple testing was performed. To evaluate the second aim of the study, comparison was undertaken between two groups and corrected for multiple testing was not required [20]. To determine the variables that predicted MCS and PCS at the final visit, two independent multivariate analysis of covariance were undertaken using age, gender and baseline MCS/PCS scores, and markers of disease severity at baseline (estimated glomerular filtration rate, eGFR, baseline TKV/Mayo subclass). The statistical significance was defined as a *P* value <0.05.

## RESULTS

### Data completion

The baseline demographic characteristics of the study population have been described elsewhere [11]. Overall, the KDQOL-SF1.3 questionnaire was completed by 89/93 (95.7%) patients in Group A (usual water intake) and 92/94 (97.9%) patients in Group B (increased water intake) at the randomization visit, and 73/78 (93.5%) patients in Group A and 72/75 (96%) patients in Group B at the final study visit. Not all patients answered the question regarding sexual function: at the randomization visit, 64/93 (68.9%) and 65/94 (69.1%) patients answered the question in Groups A and B, respectively; and at the final visit 47/78 (60.3%) and 45/75 (60%) patients answered the question in Groups A and B, respectively.

### Longitudinal changes in HRQoL in ADPKD

The longitudinal changes in KDQOL-SF1.3 sub-scales in both groups are shown in Tables 1 and 2 and Tables S5 and S6. At randomization, in both groups, the five dimensions with the lowest scores were: energy and fatigue; general and overall health; sleep; emotional well-being; and pain. Furthermore, the median PCS and MCS were similar in the two groups (*P *> 0.05). Within each group, there were no longitudinal changes over time.

**Table 1:** Summary of kidney disease targeted and SF-36 sub-scales in Group A (usual water intake) at Years 0, 1, 2, and 3.

Parameter	Year 0 *N* = 89	Year 1 *N* = 67	Year 2 *N* = 67	Year 3 *N* = 73	*P*
Kidney disease targeted sub-scales					
Symptom/problem list	86.1 ± 13.7	85.6 ± 13.9	87.7 ± 12.1	86.8 ± 12.6	0.95
Effects of kidney disease	91.7 ± 13.1	92.2 ± 13.3	92.2 ± 11.6	91.1 ± 13.9	0.98
Burden of kidney disease	83.5 ± 20.9	82.8 ± 21.8	83.2 ± 21.7	81.3 ± 24.9	0.99
Work status	83.7 ± 29.0	83.6 ± 29.6	78.5 ± 33.0	78.8 ± 33.2	0.64
Cognitive function	83.6 ± 17.8	86.9 ± 15.1	83.0 ± 19.8	86.5 ± 15.4	0.79
Quality of social interaction	80.8 ± 15.8	79.9 ± 14.9	77.9 ± 17.5	80.0 ± 16.4	0.73
Sexual function	91.2 ± 18.9 (n = 64)	94.9 ± 13.3 (n = 42)	89.8 ± 20.6 (n = 48)	94.1 ± 15.4 (n = 47)	0.37
Sleep	66.9 ± 18.1	69.5 ± 19.7	68.1 ± 18.4	67.9 ± 17.1	0.77
Social support	79.0 ± 24.9	78.5 ± 28.9	83.3 ± 20.0	84.0 ± 21.9	0.53
Overall health	71.8 ± 15.5	73.6 ± 15.9	71.5 ± 15.8	69.6 ± 18.2	0.59
SF-36 Scales					
Physical functioning	89.2 ± 16.2	85.5 ± 20.6	82.6 ± 23.4	83.8 ± 22.5	0.34
Role-physical	82.0 ± 33.2	78.4 ± 36.6	79.1 ± 36.0	80.1 ± 35.3	0.94
Pain	76.2 ± 24.1	76.2 ± 22.9	76.4 ± 21.0	75.6 ± 21.8	0.97
General health	58.7 ± 21.2	61.8 ± 20.7	57.8 ± 20.6	55.7 ± 22.2	0.26
Emotional well-being	74.4 ± 18.3	73.7 ± 19.4	74.2 ± 18.5	77.3 ± 17.5	0.51
Role-emotional	83.9 ± 31.8	82.1 ± 32.5	78.6 ± 34.2	81.3 ± 35.1	0.64
Social function	82.4 ± 21.3	84.7 ± 20.4	79.5 ± 24.9	83.7 ± 21.6	0.75
Energy fatigue	56.7 ± 19.7	55.4 ± 21.3	55.1 ± 22.3	57.8 ± 21.3	0.81
SF-12 Physical composite score	50.2 ± 7.5	48.5 ± 8.6	49.2 ± 8.3	48.8 ± 9.3	0.65
SF-12 Mental composite score	49.4 ± 9.8	49.4 ± 10.5	48.5 ± 10.9	50.2 ± 10.0	0.70

Data expressed as mean ± standard deviation. Comparisons between groups were by made by Kruskal–Wallis one-way ANOVA. *P* values are reported with significance set at *P *< 0.05.

**Table 2:** Summary of kidney disease targeted and SF-36 sub-scales in Group B (increased water intake) at Years 0, 1, 2,, and 3.

Parameter	Year 0 *N* = 92	Year 1 *N* = 62	Year 2 *N* = 61	Year 3 *N* = 72	*P*
Kidney disease targeted sub-scales					
Symptom/problem list	87.9 ± 9.5	88.1 ± 11.8	88.6 ± 11.9	88.0 ± 11.9	0.59
Effects of kidney disease	94.5 ± 7.5	94.1 ± 7.7	92.4 ± 10.7	93.4 ± 9.5	0.78
Burden of kidney disease	84.0 ± 20.3	86.6 ± 15.2	85.6 ± 19.7	84.0 ± 20.7	0.96
Work status	90.8 ± 22.2	94.2 ± 20.8	89.8 ± 24.2	88.9 ± 25.5	0.44
Cognitive function	86.7 ± 13.9	86.7 ± 14.1	86.2 ± 16.5	85.8 ± 14.2	0.94
Quality of social interaction	81.2 ± 12.9	80.3 ± 14.0	82.7 ± 13.5	80.0 ± 13.8	0.69
Sexual function	93.1 ± 17.0 (n = 65)	94.3 ± 15.1 (n = 44)	95.1 ± 11.1 (n = 38)	93.9 ± 14.5 (n = 45)	0.94
Sleep	67.9 ± 15.4	67.7 ± 17.5	66.6 ± 17.9	66.3 ± 18.8	0.94
Social support	81.0 ± 23.3	85.0 ± 22.9	83.9 ± 22.1	86.1 ± 19.2	0.38
Overall health	76.7 ± 12.3	74.3 ± 12.9	74.6 ± 13.4	72.4 ± 14.2	0.19
SF-36 Scales					
Physical functioning	87.7 ± 22.1	89.1 ± 19.5	90.5 ± 17.5	91.1 ± 14.2	0.96
Role-physical	85.6 ± 31.7	85.5 ± 30.2	84.4 ± 33.3	81.25 ± 34.0	0.83
Pain	81.2 ± 21.0	82.9 ± 18.7	83.3 ± 19.1	85.3 ± 16.7	0.78
General health	63.0 ± 18.7	63.5 ± 20.1	62.4 ± 21.5	61.4 ± 20.2	0.98
Emotional well-being	77.9 ± 13.7	77.5 ± 15.8	80.3 ± 12.8	78.4 ± 14.7	0.80
Roleeemotional	87.0 ± 27.0	87.6 ± 27.2	83.0 ± 33.1	82.9 ± 33.1	0.89
Social function	87.8 ± 17.7	88.1 ± 18.7	87.3 ± 18.5	86.8 ± 18.4	0.96
Energy fatigue	59.7 ± 18.1	57.7 ± 21.0	60.4 ± 20.1	58.6 ± 20.9	0.91
SF-12 Physical composite score	51.1 ± 7.6	51.3 ± 7.1	51.4 ± 6.7	51.3 ± 7.6	0.96
SF-12 Mental composite score	51.1 ± 8.10	50.9 ± 8.4	51.3 ± 8.4	50.6 ± 8.5	0.94

Data expressed as mean ± standard deviation. Comparisons between groups were by made by Kruskal–Wallis one-way ANOVA. *P* values are reported with significance set at *P *< 0.05.

### Effect of increased water intake on HRQoL in ADPKD

Evaluating the two groups at the final visit, the PCS was higher in Group B (48.3 ± 9.3) compared to Group A (51.3 ± 7.6; *P *= 0.037) whereas the MCS was similar between groups (Table 3 and Table S7). The improvement in the PCS was due to higher sub-scale values for physical functioning (Group B: 91.1 ± 14.2 vs. Group A: 83.8 ± 22.5; *P *= 0.04) and pain (Group B: 85.3 ± 16.7 vs. Group A: 75.6 ± 21.8; *P *= 0.0038). By multivariate analysis, the final PCS was associated with the baseline PCS and baseline TKV (Table 4). Similarly, by multivariate analysis, final MCS was associated with baseline MCS score and age, whereas baseline eGFR had a relatively minor contributing effect. In both analyses, treatment allocation (that is Group A vs. Group B) was not associated with either the PCS or MCS at the final study visit, and this result was not altered when Mayo subclass was used in the models instead of age and TKV (data not shown).

**Table 3:** Summary of kidney disease targeted and SF-36 sub-scales at the initial and final study visits in the two intervention arms.

	Initial study visit			Final study visit		
Parameter	Group A: usual water intake	Group B: increased water intake	*P*	Group A: usual water intake	Group B: increased water intake	*P*
Kidney disease targeted sub-scales						
Symptom/problem list	86.1 ± 13.7	87.9 ± 9.5	0.61	86.8 ± 12.6	88.0 ± 11.9	0.52
Effects of kidney disease	91.7 ± 13.1	94.5 ± 7.5	0.68	91.1 ± 13.9	93.4 ± 9.5	0.78
Burden of kidney disease	83.5 ± 20.9	84.0 ± 20.3	0.84	81.3 ± 24.9	84.0 ± 20.7	0.95
Work status	83.7 ± 29.0	90.8 ± 22.2	0.07	78.8 ± 33.2	88.9 ± 25.5	0.04
Cognitive function	83.6 ± 17.8	86.7 ± 13.9	0.43	86.5 ± 15.4	85.8 ± 14.2	0.53
Quality of social interaction	80.8 ± 15.8	81.2 ± 12.9	0.73	80.0 ± 16.4	80.0 ± 13.8	0.65
Sexual function	91.2 ± 18.9 (n = 64)	93.1 ± 17.0 (n = 65)	0.62	94.1 ± 15.4 (n = 47)	93.9 ± 14.5 (n = 45)	0.48
Sleep	66.9 ± 18.1	67.9 ± 15.4	0.93	67.9 ± 17.1	66.3 ± 18.8	0.56
Social support	79.0 ± 24.9	81.0 ± 23.3	0.69	84.0 ± 21.9	86.1 ± 19.2	0.81
Overall health	71.8 ± 15.5	76.7 ± 12.3	0.04	69.6 ± 18.2	72.4 ± 14.2	0.56
SF-36 Scales						
Physical functioning	89.2 ± 16.2	87.7 ± 22.1	0.60	83.8 ± 22.5	91.1 ± 14.2	0.04
Role-physical	82.0 ± 33.2	85.6 ± 31.7	0.63	80.1 ± 35.3	81.25 ± 34.0	0.97
Pain	76.2 ± 24.1	81.2 ± 21.0	0.11	75.6 ± 21.8	85.3 ± 16.7	0.0038
General health	58.7 ± 21.2	63.0 ± 18.7	0.22	55.7 ± 22.2	61.4 ± 20.2	0.08
Emotional well-being	74.4 ± 18.3	77.9 ± 13.7	0.31	77.3 ± 17.5	78.4 ± 14.7	0.91
Role-emotional	83.9 ± 31.8	87.0 ± 27.0	0.67	81.3 ± 35.1	82.9 ± 33.1	0.94
Social function	82.4 ± 21.3	87.8 ± 17.7	0.10	83.7 ± 21.6	86.8 ± 18.4	0.39
Energy fatigue	56.7 ± 19.7	59.7 ± 18.1	0.26	57.8 ± 21.3	58.6 ± 20.9	0.95
SF-12 PCS	50.2 ± 7.5	51.1 ± 7.6	0.23	48.8 ± 9.3	51.3 ± 7.6	0.037
SF-12 MCS	49.4 ± 9.8	51.1 ± 8.10	0.33	50.2 ± 10.0	50.6 ± 8.5	0.83

Data expressed as mean ± standard deviation. Comparisons between groups were by made by Wilcoxon rank sum test. *P* values are reported with significance set at *P *< 0.05.

**Table 4:** Multivariate analysis of baseline variables predicting the final PSC or final MSC scores.

	Predictors of final PCS score in model					Predictors of final MCS score in model			
Parameters	Estimate	SE	*t* ratio	*P*	Parameters	Estimate	SE	*t* ratio	*P*
Age	−0.02	0.07	−0.30	0.77	Age	0.22	0.08	2.87	0.0048
Sex (F)	−0.66	0.66	−1.00	0.32	Gender (F)	−0.53	0.72	−0.74	0.46
Treatment Group (A)	−1.13	0.64	−1.76	0.08	Treatment Group (A)	−0.04	0.70	−0.74	0.46
Baseline eGFR	0.08	0.04	1.92	0.06	Baseline eGFR	0.11	0.047	2.29	0.02
Baseline TKV	−0.002	0.001	−2.01	0.0469	Baseline TKV	0.0007	0.001	0.60	0.55
Baseline PCS	0.46	0.09	5.03	<0.0001	Baseline MCS	0.43	0.08	5.53	<0.0001

## DISCUSSION

Each person with ADPKD has a unique life-time journey punctuated by multiple varied clinical events that progressively increase in frequency and severity with ageing [21]. Owing to the chronic nature of ADPKD, it is imperative that the effects of new treatments (whether they are lifestyle or pharmacological interventions) on patient-reported measurements and HRQoL are evaluated in clinical trials. In fact, positive impacts on HRQoL are probably the key determinant of real-world treatment effectiveness, as it is likely to govern treatment acceptance and therefore the extent of uptake and long-term adherence by patients [22]. Therefore, as emphasized by others, changes in HRQoL are just as important as standard disease-specific biomarkers [4]. In the current study, the longitudinal effects of increased water intake in the PREVENT-ADPKD trial were investigated [21]. The results of the current study demonstrate that the intervention of increasing water intake did not have negative effects on emotional, mental, and physical well-being [21].

The current study is notable as being the largest cohort in which the long-term impact on increasing water intake on HRQoL has been evaluated. To our knowledge, the effect of increased water intake on HRQoL has only been assessed in one other study [23]. In a randomized controlled trial of men (aged 55 to 75 years; *n* = 140) in the community, Spigt *et al.* reported that advice to increase water intake by 1.5 l/day did not alter HRQoL (as determined by serial changes in the SF-36) compared to placebo medication (8 ml inactive syrup per day) after 6 months [23]. The intervention to increase water intake in the PREVENT-ADPKD trial was more resource-intensive than the study by Spigt *et al.*, and HRQoL was evaluated over a 3-year period. Furthermore, as kidney failure is a threatened complication, we hypothesized that participants ADPKD could experience anxiety if they were unable to reach a prescribed water target. On the other hand, participants in the intervention group in the PREVENT-ADPKD study had more frequent contact with study staff, which could have had positive benefits on HRQoL. Although the current results indicate that the intervention did not have an impact on HRQoL, the discordance between HRQoL and the declining ability to tolerate the intervention life-long (9% at 6 months to 21% at 3 years, as reported in Table S22 of reference [11]) suggests that participants probably found that increasing water intake was difficult to implement at all times, but that this did not negatively affect their general well-being.

Interestingly, the results of our study showed that the SF-12 PCS was higher in the increased water intake group compared to the usual intake group at the 3 year timepoint. This was due to improvements in SF-36 sub-scales for pain and physical functioning. Although the multivariate analysis did not find that treatment allocation (i.e. Group A vs. Group B) was associated with the final PCS, this finding is consistent with the primary results of the PREVENT-ADPKD trial, which found showing that kidney pain was lower in the increased water intake group at 18 month and 3 year timepoints (Table S20 in ref. [11]). Taken together, these data suggest that pain and physical functioning could be improved by increased water intake in ADPKD (as found in other non-renal conditions [24, 25]). This exploratory hypothesis requires further investigation in future studies in ADPKD.

HRQoL in ADPKD has only been assessed in a limited number of previous studies, mostly using the KDQOL-SF1.3 instrument [9, 19, 26–28]. In addition, only a few studies have evaluated the impact of a therapeutic intervention on HRQoL in ADPKD [9, 19]. Consistent with previous data, our results showed that the sub-scale SF-36 scores of the KDQOL-SF1.3 were similar to those of the general population [29]. This is not a surprising finding, as Miskulin and colleagues previously reported that most patients with stages 1 to 3 chronic kidney disease due to ADPKD experienced clinical symptoms, without affecting their daily living, either because symptoms were mild and/or behavioural adaptation and resilience mechanisms were activated [19]. Additionally, these data also indicate that instruments measuring HRQoL need to be purpose-built for patients with ADPKD [19, 30].

The current results provide further understanding of the pattern of changes in HRQoL in patients with ADPKD. Our results showed that energy and fatigue; general and overall health; sleep; emotional well-being; and pain were the indices that scored the lowest, as also shown in a recent large European cohort study of 465 ADPKD patients [31]. In addition, an important qualitative study of 80 patients with stage 1–2 chronic kidney disease due ADPKD showed that the majority (88%) experienced pain, breathlessness, weakness, and fatigue [4]. Of significance, these non-specific physical symptoms tend to be overlooked by healthcare providers [4]. Similarly, De Barros *et al.* reported that emotional function and general health perception were also the lowest scores in patients with ADPKD [26]. Furthermore, Miskulin *et al.* reported that patients with eGFRs between 20 and 44 ml/min/1.73 m^2^ were more likely to have greater day-to-day impact on pain and had lower SF-36 scores than those with preserved kidney function [19].

There are several limitations of the current study. First, the overall results regarding HRQoL may not have been representative of the real-world population due to selection bias associated with the inclusion and exclusion criteria of a clinical trial [32]. Second, the intervention may have had day-to-day effects that were too subtle to be detected or measured by the KDQOL-SF1.3 instrument, as suggested previously [19, 30]. Third, we used only one type of measurement to assess the impact of the intervention on HRQoL yet even completing this questionnaire extended the duration of study visits and increased the burden of the trial on participants. Thus, shorter and ADPKD-specific to instruments are needed and these should be incorporated as standard endpoints in clinical trials. Fourth, only two-thirds of participants (between 60% and 69%) completed the questions regarding sexual functioning, and hence these results should be interpreted with caution; clearly, given the paucity of data on this topic in ADPKD, further studies are needed. Finally, data on total liver volume was not available in this study and therefore the impact of severe polycystic liver disease could not be assessed.

In conclusion, the results of this study demonstrate that an intervention to increase water intake, as implemented in the PREVENT-ADPKD trial, was not associated with detrimental changes in HRQoL. Rather, the improvements in pain and physical functioning suggest possible positive effects that should be investigated in future studies in ADPKD.

## Supplementary Material

sfae159_Supplemental_File

## Data Availability

The data underlying this article will be shared on reasonable request to the corresponding author.
