# Supplementary material for: Long-term effect of increasing water intake on repeated self-assessed health-related quality of life (HRQoL) in autosomal dominant polycystic kidney disease
Source: Clin Kidney J. 2024 Jun 7;17(7):sfae159. doi: 10.1093/ckj/sfae159 (PMC11333960; doi:10.1093/ckj/sfae159)
Supplement: sfae159_Supplemental_File [file sfae159_Supplemental_File.docx]

**Table S5:** Summary of kidney disease targeted and SF-36 subscales in Group A (usual water intake) at Year 0, 1, 2 and 3.

| **Parameter** | **Year 0**  **N=89** | **Year 1**  **N=67** | **Year 2**  **N=67** | **Year 3**  **N=73** | **P** |
| --- | --- | --- | --- | --- | --- |
| **Kidney Disease Targeted Sub-Scales** |  |  |  |  |  |
| Symptom/Problem List | 88.6 (81.8-95.5) | 90.9 (79.6-95.5) | 88.6 (79.6-95.5) | 90.9 (81.8-95.5) | 0.95 |
| Effects of kidney disease | 96.9 (87.5-100) | 96.9 (90.6-100) | 96.9 (90.6-100) | 96.9 (87.5-100) | 0.98 |
| Burden of kidney disease | 93.8 (75-100) | 93.8 (68.8-100) | 93.8 (68.8-100) | 93.8 (75-100) | 0.99 |
| Work status | 100 (50-100) | 100 (50-100) | 100 (50-100) | 100 (50-100) | 0.64 |
| Cognitive function | 86.7 (73.3-100) | 86.7 (80-100) | 86/7 (73.3-100) | 93.3 (80-100) | 0.79 |
| Quality of social interaction | 86.7 (76.7-93.3) | 80 (66.7-93.3) | 80 (66.7-93.3) | 80 (73.3-93.3) | 0.73 |
| Sexual function | 100 (90.6-100)  (n=64) | 100 (100-100)  (n=42) | 100 (87.5-100)  (n=48) | 100 (100-100)  (n=47) | 0.37 |
| Sleep | 67.5 (52.5-80) | 72.5 (57.5-83.8) | 72.5 (55-82.5) | 70 (55-82.5) | 0.77 |
| Social support | 83.3 (66.7-100) | 83.3 (66.7-100) | 83.3 (66.7-100) | 100 (66.7-100) | 0.53 |
| Overall health | 70 (60-80) | 80 (70-80) | 70 (60-80) | 70 (60-80) | 0.59 |
| **SF-36 Scales** |  |  |  |  |  |
| Physical functioning | 95 (85-100) | 95 (80-100) | 95 (95-100) | 95 (77.5-100) | 0.34 |
| Role-Physical | 100 (75-100) | 100 (75-100) | 100 (75-100) | 100 (75-100) | 0.94 |
| Pain | 80 (67.5-90) | 77.5 (67.5-90) | 80 (67.5-90) | 90 (77.5-100) | 0.97 |
| General health | 65 (45-75) | 65 (50-75) | 55 (45-70) | 55 (40-70) | 0.26 |
| Emotional well-being | 80 (64-88) | 76 (64-88) | 76 (64-88) | 84 (66-92) | 0.51 |
| Role – Emotional | 100 (83.3-100) | 100 (66.7-100) | 100 (66.7-100) | 100 (83.3-100) | 0.64 |
| Social function | 100 (75-100) | 100 (75-100) | 87.5 (62.5-100) | 87.5 (75-100) | 0.75 |
| Energy fatigue | 55 (42.5-72.5) | 55 (45-75) | 55 (40-70) | 60 (45-75) | 0.81 |
| SF-12 Physical composite score | 52.5 (46.6-55.5) | 51.9 (44.3-54.7) | 52.5 (43.4-55.5) | 50.4 (45.7-55.5) | 0.65 |
| SF-12 Mental composite score | 52.2 (42.4-57.1) | 53.3 (45.0-57.1) | 51.3 (41.0-57.8) | 54.7 (44.7-57.5) | 0.70 |

Data expressed as median (quartile 1 to quartile 3). Comparisons between groups were by made by Kruskal Wallis one-way ANOVA. P values are reported with significance set at P<0.05.

**Table S6:** Summary of kidney disease targeted and SF-36 subscales in Group B (increased water intake) at Year 0, 1, 2 and 3.

| **Parameter** | **Year 0**  **N=92** | **Year 1**  **N=62** | **Year 2**  **N=61** | **Year 3**  **N=72** | **P** |
| --- | --- | --- | --- | --- | --- |
| **Kidney Disease Targeted Sub-Scales** |  |  |  |  |  |
| Symptom/Problem List | 90.5 (84.1-93.2) | 90.9 (84.1-96.0) | 93.2 (82.2-97.7) | 90.9 (81.8-97.7) | 0.59 |
| Effects of kidney disease | 96.9 (92.9-100) | 96.9 (90.6-100) | 96.9 (90.6-100) | 96.9 (90.6-100) | 0.78 |
| Burden of kidney disease | 93.8 (75-100) | 93.8 (68.8-100) | 93.8 (81.3-100) | 93.8 (75-100) | 0.96 |
| Work status | 100 (100-100) | 100 (100-100) | 100 (100-100) | 100 (100-100) | 0.44 |
| Cognitive function | 86.7 (80-100) | 86.7 (85.0-100) | 93.3 (81.7-100) | 90 (80-100) | 0.94 |
| Quality of social interaction | 80 (73.3-93.3) | 86.7 (73.3-88.3) | 80 (75.0-93.3) | 80 (66.7-93.3) | 0.69 |
| Sexual function | 100 (100-100)  (n=65) | 100 (100-100)  (n=44) | 100 (100-100)  (n=38) | 100 (93.8-100)  (n=45) | 0.94 |
| Sleep | 70 (57.5-77.5) | 72.5 (57.5-80) | 71.3 (53.1-79.4) | 67.5 (50-84.4) | 0.94 |
| Social support | 83.3 (66.7-100) | 100 (66.7-100) | 100 (66.7-100) | 100 (83.3-100) | 0.38 |
| Overall health | 80 (70-87.5) | 80 (70-80) | 80 (70-80) | 70 (60-80) | 0.19 |
| **SF-36 Scales** |  |  |  |  |  |
| Physical functioning | 95 (90-100) | 95 (88.8-100) | 97.5 (90-100) | 97.5 (86.25-100) | 0.96 |
| Role-Physical | 100 (100-100) | 100 (93.8-100) | 100 (100-100) | 100 (75-100) | 0.83 |
| Pain | 90 (70-100) | 90 (75.6-100) | 90 (70-100) | 90 (77.5-100) | 0.78 |
| General health | 65 (50-75) | 65 (50-80) | 70 (47.5-77.5) | 65 (50-75) | 0.98 |
| Emotional well-being | 84 (68-88) | 84 (72-88) | 84 (74-90) | 84 (68-88) | 0.80 |
| Role – Emotional | 100 (100-100) | 100 (100-100) | 100 (83.3-100) | 100 (75.0-100) | 0.89 |
| Social function | 100 (75-100) | 100 (75-100) | 100 (75-100) | 100 (75-100) | 0.96 |
| Energy fatigue | 60 (50-73.8) | 60 (45-75) | 65 (40-78.75) | 60 (41.3-78.8) | 0.91 |
| SF-12 Physical composite score | 53.8 (49.2-55.5) | 53.2 (48.0-55.9) | 54.1 (49.5-55.7) | 54.1 (50.0-55.9) | 0.96 |
| SF-12 Mental composite score | 53.6 (47.6-57.1) | 53.3 (47.5-56.7) | 54.7 (46.8-57.8) | 53.3 (45.6-58.8) | 0.94 |

Data expressed as median (quartile 1 to quartile 3). Comparisons between groups were by made by Kruskal Wallis one-way ANOVA. P values are reported with significance set at P<0.05.

**Table S7:** Summary of kidney disease targeted and SF-36 subscales at the initial and final study visits in the two intervention arms.

| **Parameter** | **Initial Study Visit** | | **P** | **Final Study Visit** | | **P** |
| --- | --- | --- | --- | --- | --- | --- |
|  | Group A: Usual Water Intake | Group B: Increased Water intake |  | Group A: Usual Water Intake | Group B: Increased  Water intake |  |
| **Kidney Disease Targeted Sub-Scales** |  |  |  |  |  |  |
| Symptom/Problem List | 88.6 (81.8-95.5) | 90.5 (84.1-93.2) | 0.61 | 90.9 (81.8-95.5) | 90.9 (81.8-97.7) | 0.52 |
| Effects of kidney disease | 96.9 (87.5-100) | 96.9 (92.9-100) | 0.68 | 96.9 (87.5-100) | 96.9 (90.6-100) | 0.78 |
| Burden of kidney disease | 93.8 (75-100) | 93.8 (75-100) | 0.84 | 93.8 (75-100) | 93.8 (75-100) | 0.95 |
| Work status | 100 (50-100) | 100 (100-100) | 0.07 | 100 (50-100) | 100 (100-100) | 0.04 |
| Cognitive function | 86.7 (73.3-100) | 86.7 (80-100) | 0.43 | 93.3 (80-100) | 90 (80-100) | 0.53 |
| Quality of social interaction | 86.7 (76.7-93.3) | 80 (73.3-93.3) | 0.73 | 80 (73.3-93.3) | 80 (66.7-93.3) | 0.65 |
| Sexual function | 100 (90.6-100)  (n=64) | 100 (100-100)  (n=65) | 0.62 | 100 (100-100)  (n=47) | 100 (93.8-100)  (n=45) | 0.48 |
| Sleep | 67.5 (52.5-80) | 70 (57.5-77.5) | 0.93 | 70 (55-82.5) | 67.5 (50-84.4) | 0.56 |
| Social support | 83.3 (66.7-100) | 83.3 (66.7-100) | 0.69 | 100 (66.7-100) | 100 (83.3-100) | 0.81 |
| Overall health | 70 (60-80) | 80 (70-87.5) | 0.04 | 70 (60-80) | 70 (60-80) | 0.56 |
| **SF-36 Scales** |  |  |  |  |  |  |
| Physical functioning | 95 (85-100) | 95 (90-100) | 0.60 | 95 (77.5-100) | 97.5 (86.25-100) | 0.04 |
| Role-Physical | 100 (75-100) | 100 (100-100) | 0.63 | 100 (75-100) | 100 (75-100) | 0.97 |
| Pain | 80 (67.5-90) | 90 (70-100) | 0.11 | 90 (77.5-100) | 90 (77.5-100) | 0.0038 |
| General health | 65 (45-75) | 65 (50-75) | 0.22 | 55 (40-70) | 65 (50-75) | 0.08 |
| Emotional well-being | 80 (64-88) | 84 (68-88) | 0.31 | 84 (66-92) | 84 (68-88) | 0.91 |
| Role – Emotional | 100 (83.3-100) | 100 (100-100) | 0.67 | 100 (83.3-100) | 100 (75.0-100) | 0.94 |
| Social function | 100 (75-100) | 100 (75-100) | 0.10 | 87.5 (75-100) | 100 (75-100) | 0.39 |
| Energy fatigue | 55 (42.5-72.5) | 60 (50-73.8) | 0.26 | 60 (45-75) | 60 (41.3-78.8) | 0.95 |
| SF-12 Physical composite score | 52.5 (46.6-55.5) | 53.8 (49.2-55.5) | 0.23 | 50.4 (45.7-55.5) | 54.1 (50.0-55.9) | 0.037 |
| SF-12 Mental composite score | 52.2 (42.4-57.1) | 53.6 (47.6-57.1) | 0.33 | 54.7 (44.7-57.5) | 53.3 (45.6-58.8) | 0.83 |

Data expressed as median (quartile 1 to quartile 3). Comparisons between groups were by made by Wilcoxon Rank Sum test. P values are reported with significance set at P<0.05.
